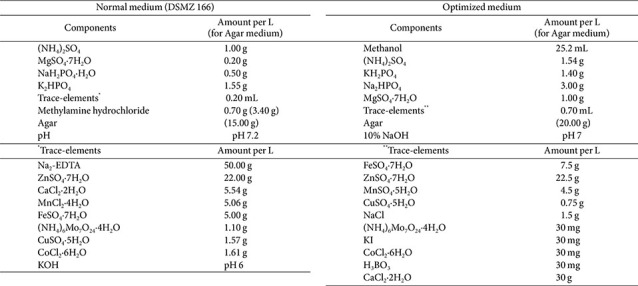# Optimization of Culture Conditions for Pyrroloquinoline Quinone-Overproducing Mutant *Hyphomicrobium denitrificans* and Its Skin Bioactive Properties

**DOI:** 10.4014/jmb.2603.03038

**Published:** 2026-05-08

**Authors:** Jiwon Lee, Hyunjoo Hwang, Gamin Kim, Peter Hinterdorfer, Soyoun Song, Kisung Ko

**Affiliations:** 1 BioSystems Design Lab, Department of Medicine, College of Medicine, Chung-Ang University, Seoul 06974, Republic of Korea; 2 Novepharma Inc., Yongin 17095, Republic of Korea; 3 Department of Applied Experimental Biophysics, Institute of Biophysics, Johannes Kepler University Linz 4040 Linz, Austria

**Keywords:** Pyrroloquinoline quinone (PQQ), Mutant strain, Optimized culture conditions, Skin physiology

## Abstract

Pyrroloquinoline quinone (PQQ) is a water-soluble quinone derived from bacteria, known for its antioxidant properties and ability to enhance mitochondrial function. These characteristics make PQQ a promising bioactive molecule for anti-aging applications in dermatology. This study aimed to improve PQQ production by developing a UV-induced mutant strain and optimizing the culture medium, and to evaluate the potential of the produced PQQ as a cosmetic ingredient. The mutant strain, NPP-230, was compared with the wild-type strain, *Hyphomicrobium denitrificans* ATCC51888, for growth and PQQ production after 96 h of cultivation. The effect of optimized medium on both strains was also assessed. The PQQ obtained from the cultures was evaluated for skin-related biological activities, including collagen synthesis, melanin inhibition, tyrosinase inhibition, and L-3,4-dihydroxyphenylalanine (L-DOPA) oxidation inhibition. The mutant strain cultured in optimized medium showed higher biomass and PQQ production than the wild type. Furthermore, PQQ promoted collagen synthesis and inhibited melanin production and tyrosinase activity in a concentration-dependent manner. These findings indicate that PQQ production in *H. denitrificans* can be enhanced under optimized conditions, and that the produced PQQ exhibits anti-aging and skin-whitening properties.

## Introduction

**Fig. 1:**
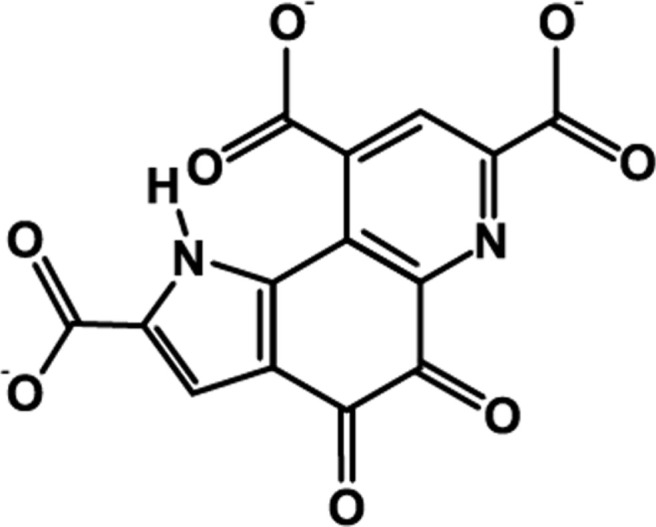
PQQ exhibits a unique heterocyclic structure characterized as 4,5-dioxo-4,5-dihydro-1H-pyrrolo[2,3-f]quinoline-2,7,9- tricarboxylic acid.

Pyrroloquinoline quinone (PQQ) is a redox-active quinone compound first identified in methanol-utilizing bacteria and initially termed “methoxatin” [1] (Fig. 1). It was later recognized as a novel cofactor structurally distinct from Nicotinamide Adenine Dinucleotide (NAD^+^) and Flavin Adenine Dinucleotide (FAD), playing essential roles in microbial alcohol and glucose dehydrogenases [2]. Beyond microbial systems, PQQ has been detected in various dietary sources such as parsley, soybeans, potatoes, kiwifruit, and human breast milk [2, 3]. PQQ has been proposed as a conditionally essential nutrient. However, this classification remains under debate [4, 5]. A growing body of evidence highlights the diverse biological activities of PQQ, including potent antioxidant capacity, stimulation of mitochondrial biogenesis, and neuroprotective effects [6, 7]. It has been reported to enhance nerve growth factor (NGF) expression and improve brain function, supporting its use as a functional dietary supplement [8, 9]. In comparison with coenzyme Q10 (CoQ10), another quinone-based compound, PQQ shows distinct molecular properties and mechanisms of action [10, 11]. In recent years, interest in the cosmetic potential of PQQ has emerged. It has been shown to increase collagen levels and promote skin cell proliferation, while also reducing melanin synthesis through tyrosinase inhibition *in vitro* and in animal models [12-14]. These findings suggest that PQQ may be applicable as an active ingredient in cosmetics targeting skin health and pigmentation control [15]. However, microbial fermentation of PQQ is often limited by low yields, restricting its broader industrial application. Native PQQ-producing strains such as *H. denitrificans* (ATCC51888) exhibit low productivity under standard conditions. To address this, several studies have focused on increasing yield through UV mutagenesis, gene cloning, and metabolic pathway optimization [16, 17]. Additionally, process parameters including oxygen levels, pH, trace elements, and nutrient sources have been optimized to enhance fermentation efficiency [18]. In our previous work, a high-yield mutant strain, NPP-230 (Korea Patent No. 10-2120670), was developed using UV mutagenesis. Subsequent optimization of culture conditions—such as trace elements, phosphoric acid concentration, and carbon/nitrogen sources—further enhanced PQQ production. In this study, we evaluated the cell growth and PQQ production capacity of NPP-230 under both standard and optimized fermentation conditions and compared it to the wild-type strain *H. denitrificans* ATCC51888. Additionally, the skin-related biological activities of the biosynthesized PQQ were assessed through *in vitro* assays, including measurements of collagen synthesis, melanin inhibition, tyrosinase activity, and L-3,4-dihydroxyphenylalanine (L-DOPA) oxidation, to explore its application as a cosmetic bioactive compound.

## Materials and Methods

### Bacterial Strains and Fermentation

**Fig. 2:**
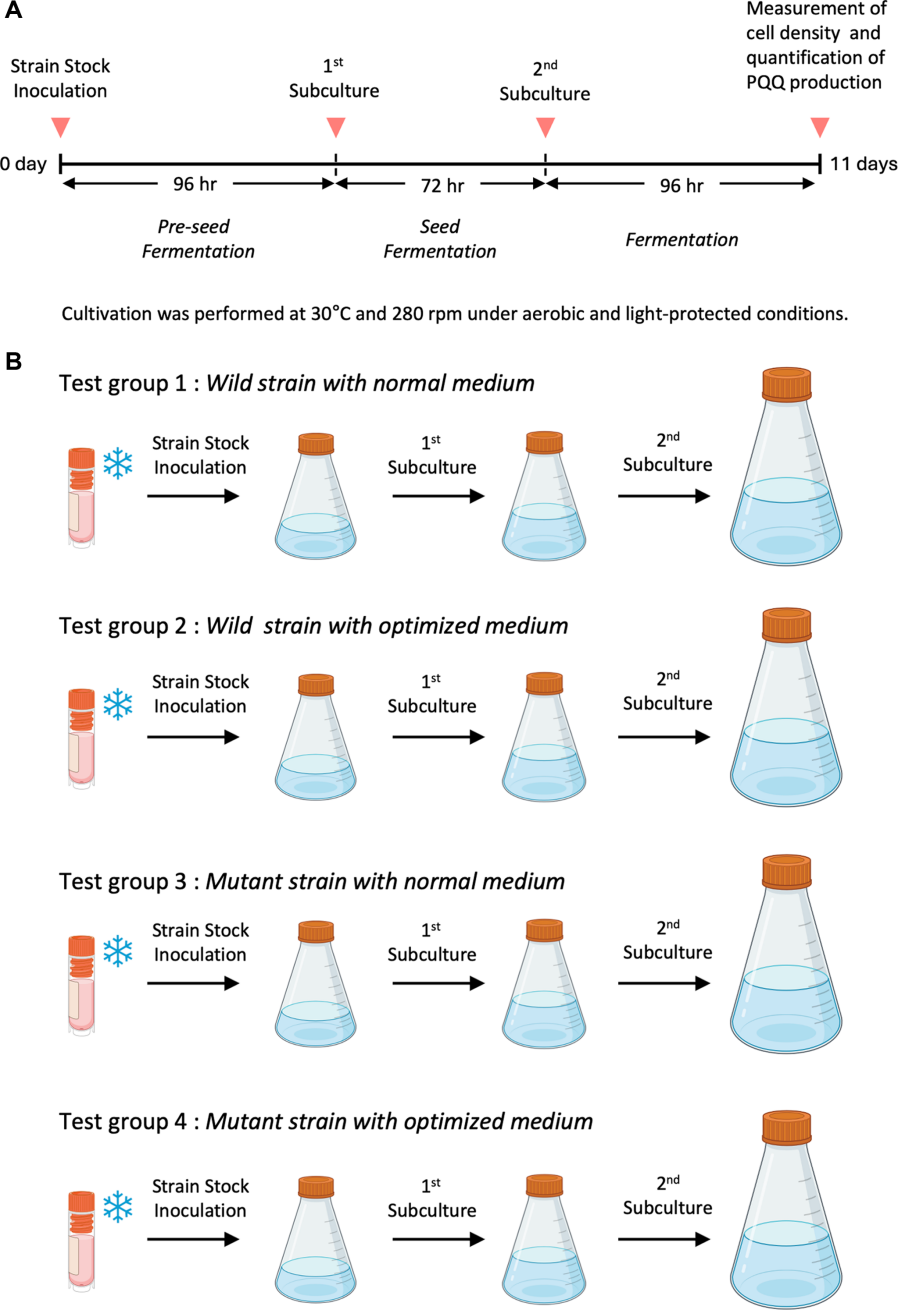
(A) Schematic diagram illustrating the three-step subculture fermentation process. (B) Cell growth groups of wild and mutant strains under normal and optimized medium conditions.

The strains used in this study were *H. denitrificans* ATCC51888 (KACC 21515) (KACC, Republic of Korea) and UV-induced mutant NPP-230 (KCTC13718BP) (Korean Patent No. 10-2120670) developed by Novepharma (Republic of Korea). The UV-mutated strain NPP-230 was generated by exposing the parental strain to UV irradiation at a wavelength of 253 nm, with exposure times of 5, 10, 20, 30, 40, 50, and 60 s at a fixed distance of 30 cm, followed by recovery in the dark to prevent photoreactivation. Mutants were then screened on culture media containing 2–5% methanol, and NPP-230 was selected for further analysis. Both strains were cryopreserved at -70°C in a 25% glycerol stock. The entire fermentation process consisted of a three-step scale-up subculture: 96 h of preseed, 72 h of seed, and 96 h of main fermentation (Fig. 2A). To ensure evenness of the biomass at subculture, a correction curve between optical density (OD_600_) and the number of viable cells [Colony Forming Unit (CFU)/mL] was established. Linear correlations are equation Log_10_ (CFU/mL) = 0.3387 × OD_600_ + 8.8068 (R^2^ = 0.978), which was used to standardize inoculum volumes. The preseed culture was performed in 15 mL of liquid medium for 96 h, followed by a seed culture in 25 mL for 72 h. The main fermentation was conducted in 500 mL working volume within 1 L baffled aerobic flasks for 96 h. At each stage, 2% (v/v) inoculum based on an OD_600_ = 1.000 was used. All culturing steps were carried out at 30°C with shaking at 280 rpm under light-blocking conditions. Cell density was monitored spectrophotometrically at 600 nm.

### Media Composition and Culture Conditions

Normal medium [1.00 g/L of (NH_4_)_2_SO_4_, 0.20 g/L of MgSO_4_·7H_2_O, 0.50 g/L of NaH_2_PO_4_·H_2_O, 1.55 g/L of K_2_HPO_4_, 0.70 g/L of methylamine hydrochloride, 0.20 mL/L of trace-element and pH 7.2] and optimized medium [1.54 g/L of (NH_4_)_2_SO_4_, 1.40 g/L of KH_2_PO_4_, 3.00 g/L of Na_2_HPO_4_, 1.00 g/L of MgSO_4_·7H_2_O, 25.2 mL/L of methanol, 0.70 mL/L of trace-element and pH 7] were used to compare cell growth and PQQ productivity of wild and mutant strains according to culture conditions (Table 1). For methanol stability, the optimization medium was supplemented with methanol immediately before inoculation, following 0.2 μm membrane filtration and sterilization. Cultivation was performed in disposable baffled flasks under light-blocked conditions at 30°C with shaking at 280 rpm for a total of 11 days through serial subcultures. Four experimental conditions were tested: wild strain with normal medium, wild strain with optimization medium, mutant strain with normal medium, and mutant strain with optimization medium (Fig. 2B). Each condition was conducted in triplicate. OD_600_ was measured after 96 h of main fermentation in 500 mL flasks to evaluate final cell density and quantification of PQQ production.

### HPLC Analysis of PQQ

After 96 h of main fermentation, the culture medium was collected to assess the PQQ content contained in the culture medium. The supernatant from each of the four experimental groups was obtained by centrifugation, and residual foreign substances were removed using a 0.2 μm membrane filter. The filtrate was appropriately diluted with distilled water to ensure that the PQQ concentration fell within the calibration range, and an aliquot (10 μL) was directly injected into the HPLC system. The concentration of PQQ in the resulting filtrate was measured using high-performance liquid chromatography (HPLC). Separation was carried out on a reverse-phase YMC-Triart C18 column (4.6 × 250 mm, 5 μm). Standard PQQ (Sigma, USA) was prepared by diluting in distilled water to final concentrations of 1.67–26.7 μM. Quantitative analysis of PQQ was performed using an external standard calibration curve. Standard solutions of PQQ were analyzed under the same HPLC conditions. The calibration curves showed linearity, with regression equations of y = 35629x – 10977 (R^2^ = 1.0000) at 250 nm. All sample concentrations were within the calibration range. UV absorbance was measured at 250 nm, and quantitative analysis was performed through the characteristic spectral peaks of PQQ. The column was maintained at 30°C, and 10 μL was used for the sample. As the mobile-phase solvent, solvent A used 15 mM phosphate buffer (pH 7.4) and solvent B used acetonitrile. The elution program began with 97% solvent A and 3% solvent B, held isocratically for 6 min. At 6.01 min, the gradient shifted sharply to 20% solvent A and 80% solvent B, which was maintained until 27 min. At 27.01 min, the composition was returned to initial conditions (97% solvent A, 3% solvent B) and held constant until 33 min to allow for column re-equilibration.

### Cell Viability Assay in Human Dermal Fibroblasts (CCD-986sk)

Cell viability was evaluated using the EZ-Cytox Enhanced Cell Viability Assay Kit (DoGenBio, Republic of Korea). Human dermal fibroblasts (CCD-986sk) (ATCC, USA) were cultured in Iscove’s Modified Dulbecco’s Medium (IMDM; Welgene, Republic of Korea) and seeded into 96-well culture plates (SPL Life Sciences, Pocheon, Korea) at a density of 7 × 10^3^ cells per well. After incubation at 37°C in a humidified atmosphere containing 5% CO_2_ for 24 h to allow cell attachment, the culture medium was removed and the cells were washed once with 1× Dulbecco’s Phosphate-Buffered Saline (DPBS). PQQ used in this study was produced in-house via microbial fermentation using the mutant strain (NPP-230) described above, followed by purification prior to use. Cells were treated with PQQ at final concentrations of 0, 100, 250, or 500 μM prepared in IMDM and incubated for an additional 48 h under standard culture conditions (37°C, 5% CO_2_). Following treatment, the wells were washed once with 1× DPBS to remove residual compounds. A working solution of IMDM and EZ-Cytox reagent (10:1, v/v) was added to each well, and the plates were incubated for 4 h at 37°C in a CO_2_ incubator. Prior to measurement, the plates were gently shaken for 1 min to ensure homogeneous color development. Absorbance was measured at 450 nm using a microplate reader. Cell viability was expressed as relative cell viability (%), calculated by normalizing the absorbance values of PQQ-treated cells to those of the untreated control group (0 μM), which was set to 100%. Data are presented as mean ± standard deviation. Statistical significance compared with the control group is indicated in the figure (*p < 0.05; ns, not significant). All measurements were performed in triplicate. Cell viability (%) was calculated using the following equation:

$$\text{Cell Viability (\%)}=\left(\frac{{\text{OD}}_{450\text{nm}}\text{ of sample treated group}}{{\text{OD}}_{450\text{nm}}\text{ of Control group}}\right)\times 100$$

### Collagen Synthesis Assay

Collagen synthesis assay was in accordance with the MFDS guidelines for functional cosmetics efficacy evaluation. Collagen production was evaluated in human dermal fibroblasts (CCD-986sk) (ATCC, USA) to assess the bioactivity of PQQ. For the assay, cells were seeded in 48-well plates at a density of 5 × 10^4^ cells/well and allowed to adhere for 24 h. Culture medium was maintained in IMDM supplemented with 10% fetal bovine serum (FBS) and 1% penicillin-streptomycin, under standard culture conditions (37°C, 5% CO_2_, humidified incubator). After 24 h, the culture medium was removed from each well and rinsed with PBS, cells were treated with serum-free IMDM containing 1% penicillin-streptomycin, supplemented 1 mL with either L-ascorbic acid 284 μM or PQQ at concentrations of 100, 250, and 500 μM, followed by an additional 24 h incubation. After treatment, culture supernatants were harvested and analyzed for procollagen Type I C-peptide levels using Procollagen Type I C-Peptide EIA Kit (Takara Bio, Japan) following the manufacturer’s protocol. The collagen content was calculated based on a standard calibration curve established with known concentrations of procollagen. To normalize collagen production against cellular protein content, total cellular protein was extracted after removing the culture medium. Each well received 200 μL of M-PER^TM^ Mammalian Protein Extraction Reagent (Thermo Fisher Scientific, USA), followed by shaking at 150 rpm for 10 min at room temperature. The lysates were collected and centrifuged, and the resulting supernatant was used for protein quantification using the BCA Protein Assay Kit (Thermo Fisher Scientific). The protein concentration was determined using a bovine serum albumin (BSA) standard curve. Collagen synthesis was expressed as the ratio of collagen to total protein. All measurements were performed in triplicate. The relative intracellular collagen synthesis rate was calculated using the following formula:

$$\text{Collagen synthesis rate (\%)}=\left(\frac{{\text{Collagen/Protein}}_{\text{Sample}}}{{\text{Collagen/Protein}}_{\text{control}}}\right)\times 100$$

### Cell Viability Assay in B16-F10 Mouse Melanoma Cells

Cell viability assay was conducted in accordance with the Ministry of Food and Drug Safety (MFDS) (Republic of Korea) guidelines for functional cosmetics efficacy evaluation. To assess the cytotoxicity of PQQ, a cell viability assay was performed using five different concentrations: 0, 50, 100, 250, and 500 μM. PQQ was first dissolved in distilled water to prepare a 100 mM stock solution and subsequently diluted in high-glucose, phenol red-free Dulbecco’s Modified Eagle’s Medium (DMEM) supplemented with 10% FBS and 1% penicillin-streptomycin to obtain the desired final concentrations. B16-F10 mouse melanoma cells (ATCC) were seeded in 24-well plates at a density of 2.0 × 10^4^ cells/well in 500 μL of complete DMEM (10% FBS, 1% penicillin-streptomycin), and cultured under standard conditions (37°C, 5% CO_2_, humidified incubator) for 24 h. After incubation, the culture medium was removed and the cells were rinsed with PBS. Subsequently, 990 μL of fresh phenol red-free high-glucose DMEM was added to each well, followed by the addition of 10 μL of the prepared PQQ solution. The cells were then incubated for an additional 24 h. Cell viability was evaluated using the Water-Soluble Tetrazolium salt (WST) assay. WST reagent was diluted 1:10 in the culture medium and added directly to each well, followed by incubation at 37°C for 2 h. Absorbance was measured at 450 nm using a microplate reader. All measurements were performed in triplicate. Cell viability (%) was calculated using the following equation:

$$\text{Cell Viability (\%)}=\left(\frac{{\text{OD}}_{450\text{nm}}\text{ of sample treated group}}{{\text{OD}}_{450\text{nm}}\text{ of Control group}}\right)\times 100$$

### Melanin Inhibition Assay

Melanin inhibition assay was conducted in accordance with the MFDS guidelines for functional cosmetics efficacy evaluation. The melanin inhibition effect of PQQ was evaluated using B16-F10 mouse melanoma cells (ATCC). PQQ was prepared at 100 mM in distilled water and then diluted in serum-free DMEM containing 1% penicillin-streptomycin and 100 nM α-MSH to 0, 50, 100, and 250 μM. Cells were seeded in 24-well plates at a density of 2.0 × 10^4^ cells/well in 500 μL of complete DMEM, and incubated under standard culture conditions (37°C, 5% CO_2_, humidified incubator) for 24 h. After incubation, the culture medium was removed and cells were washed with PBS. Subsequently, PQQ at final concentrations of 50, 100, and 250 μM was added to each well. As controls, only 100 nM α-MSH was used as a negative control and 100 μM arbutin (included 100 nM α-MSH) as a positive control. The treated cells were then incubated for an additional 72 h. Following treatment, the culture medium was discarded, and the cells were washed again with PBS. Cells were detached using trypsin-EDTA, harvested by centrifugation at 1,000 rpm for 3 min, and resuspended in 1 N NaOH containing 10% dimethyl sulfoxide (DMSO). The suspension was heated at 80°C for 1 h to solubilize intracellular melanin. Melanin content was quantified by measuring absorbance at 475 nm, and calculated based on a standard curve constructed using synthetic melanin. In parallel, total cellular protein was quantified using the BCA Protein Assay Kit (Pierce, USA), with BSA as the standard. All measurements were performed in triplicate. The percentage of melanin synthesis inhibition was calculated using the following equation:

$$\text{Melanin Inhibition Rate (\%)}=100-\left(\frac{{\text{Melanin/Protein}}_{\text{Sample}}}{{\text{Melanin/Protein}}_{\text{Control}}}\times 100\right)$$

### Tyrosinase Inhibition Assay

Tyrosinase inhibition assay was conducted in accordance with the MFDS guidelines for functional cosmetics efficacy evaluation. Tyrosine (Sigma) (1 mM) and mushroom tyrosinase (Sigma) (0.1 U/mL) were each dissolved in PBS and used as the substrate and enzyme source, respectively. PQQ was prepared as a 10 mM stock solution in distilled water and subsequently diluted with PBS to achieve final concentrations of 50, 100, and 250 μM. 3-O-Ethyl-L-ascorbic acid (TCI, Japan) 2450 μM was used as a positive control. The assay was performed in 96-well plates. For each treatment group, 10 μL of PBS or test sample was added to 50 μL of tyrosinase (0.1 U/mL), followed by incubation at 37°C for 15 min. Then, 50 μL of either L-tyrosine (1 mM) or PBS was added depending on the experimental group. The groups were organized as follows. Reactions were initiated by mixing enzyme and substrate with the test samples or controls and incubated at 37°C for 15 min. After the incubation period, absorbance was measured at 495 nm using a microplate reader to quantify dopachrome formation, which reflects tyrosinase activity. All measurements were performed in triplicate. The percent inhibition of tyrosinase activity by PQQ was calculated using the following formula:

$$\text{Tyrosinase Inhibition Rate (\%)}=100-\left(\frac{b^{\prime }-b}{a^{\prime }-a}\times 100\right)$$

### L-DOPA Oxidation Inhibition Assay

L-DOPA oxidation inhibition assay was conducted in accordance with the MFDS guidelines for functional cosmetics efficacy evaluation. Tyrosinase (Sigma) and L-DOPA (Sigma) were each dissolved in PBS and used at final concentrations of 0.1 U/mL and 0.6 mM, respectively. PQQ was prepared as a 10 mM stock solution in distilled water and further diluted in PBS to obtain final concentrations of 50, 100, and 250 μM. 3-O-Ethyl-L-ascorbic acid (TCI) was used as a positive control at a final concentration of 2450 μM. The assay was performed in 96-well plates. For each treatment group, 10 μL of PBS or test sample was added to 50 μL of tyrosinase (0.1 U/mL), followed by incubation at 37°C for 15 min. Then, 50 μL of either L-DOPA (0.6 mM) or PBS was added depending on the experimental group. The groups were organized as follows. Reactions were initiated by mixing enzyme and substrate with the test samples or controls and incubated at 37°C for 15 min. After the reaction, absorbance was measured at 475 nm to detect dopachrome formation. All measurements were performed in triplicate. L-DOPA oxidation inhibition was calculated according to the following formula:

$$\text{L–DOPA Inhibition Rate (\%)}=100-\left(\frac{b^{\prime }-b}{a^{\prime }-a}\times 100\right)$$

### Statistical Analysis

All data are presented as mean ± standard deviation (SD). Statistical analysis was performed using one-way analysis of variance (ANOVA), followed by Dunnett’s post hoc test for comparisons with the control group. A *p*-value < 0.05 was considered statistically significant.

## Results

### Cell Growth under Wild and Mutant Strains in Different Medium

**Fig. 3:**
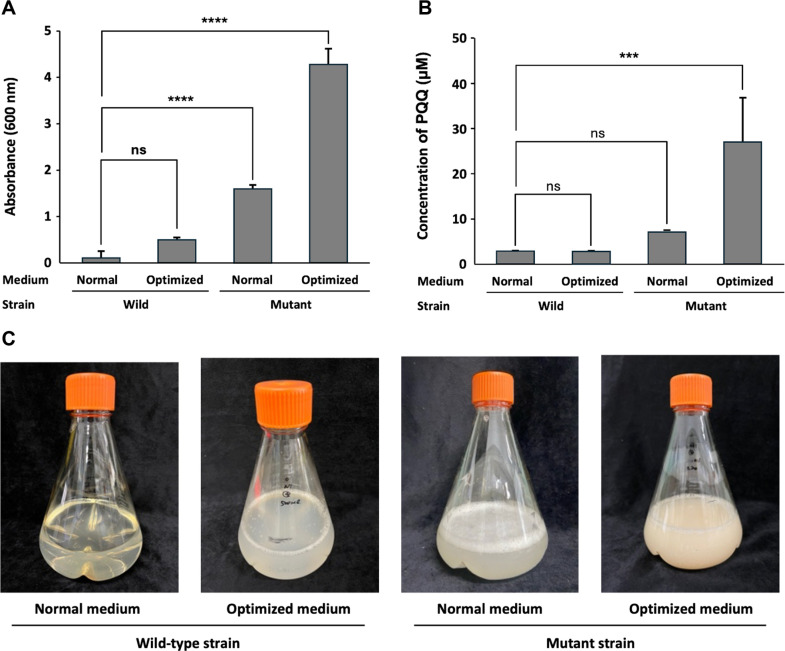
Comparison of bacterial growth and PQQ production between wild and mutant strains under normal and optimized media conditions. (**A**) Cell growth was monitored by OD_600_ measurement after 96 h of main fermentation in wild and mutant strains. (**B**) PQQ production was quantified in the culture supernatants by HPLC after 96 h of fermentation. Mutant strains produced higher PQQ concentrations than wild strains, with the highest yield obtained under optimized medium conditions. Statistical significance was determined using one-way ANOVA followed by Dunnett’s test compared to the wild-type strain cultured in normal medium (ns, not significant; *p < 0.05; **p < 0.01; ***p < 0.001; ****p < 0.0001). (**C**) Visual appearance of the 500 mL fermentation broth after 96 h. Mutant strains showed significantly enhanced cell growth compared to wild strains in both media types, with the highest OD_600_ observed in mutant strains cultured in optimized medium

This study was performed to evaluate the effects of induced mutant strains and optimized media to increase the bacterial growth and PQQ production yield. Wild and mutant strains were cultured in both normal and optimized media, and OD_600_ was measured after the main fermentation, which was performed identically for 96 h, to compare the biomass. The mutant strains showed greater cell growth than the wild strain in both medium types. In normal media, the wild strain showed an average OD_600_ of 0.108 ± 0.153, and the mutant strain showed 1.592 ± 0.079. Growth was further improved under optimized media conditions, with OD_600_ values of 0.485 ± 0.037 for the wild strain and 4.252 ± 0.334 for the mutant strain. The cell density of the mutant strain increased approximately 8.8-fold compared to the wild when cultured in the optimized medium, and approximately 39.4-fold compared to the cell density of the wild strain in the normal medium. The results of this study suggest that the mutant strain improved bacterial growth compared to the wild strain, and that the optimized medium further improved the growth of the mutant strain (Fig. 3A).

### Comparison of PQQ Production under Different Condition

To evaluate the effect of strain type and medium optimization on PQQ production, wild and mutant strains were cultured in both normal and optimized media. PQQ concentration in the culture supernatant was quantified by HPLC. According to the quantitative results, the wild strain cultivated in normal medium produced 3.06 ± 0.12 μM of PQQ, whereas a slightly reduced amount of 2.64 ± 0.10 μM was observed when the same strain was grown in the optimized formulation. On the other hand, the mutant strain yielded 7.44 ± 0.22 μM under normal conditions, with a marked increase to 28.56 ± 10.19 μM under optimized conditions. The highest PQQ production was recorded in the mutant strain cultured with the optimized medium, showing approximately a 9.3-fold improvement compared to the wild strain in normal conditions (Fig. 3B). These outcomes clearly demonstrate that the combination of genetic enhancement and medium refinement substantially elevates PQQ biosynthetic efficiency. Particularly, the combination of the mutant strain and optimized culture conditions was the most effective in boosting PQQ yield.

### Collagen Synthesis Activity of PQQ

**Fig. 4:**
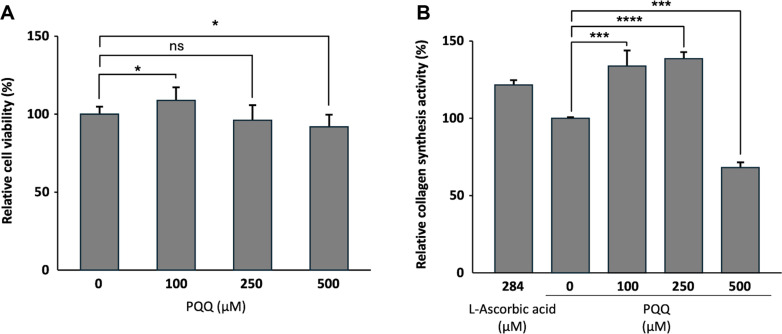
Effects of PQQ on cell viability and collagen production. (**A**) Cell viability of CCD-986sk human dermal fibroblasts treated with PQQ at various concentrations. (**B**) Effect of PQQ on collagen production in CCD-986sk cells. Cells were treated with PQQ at final concentrations of 100, 250, and 500 μM for 24 h, and intracellular collagen levels were quantified. PQQ treatment increased collagen production at 100 and 250 μM, whereas a decrease was observed at 500 μM. Statistical significance was determined using one-way ANOVA followed by Dunnett’s test compared with the untreated control group (ns, not significant; *p < 0.05; **p < 0.01; ***p < 0.001; ****p < 0.0001).

To evaluate the effect of PQQ on collagen synthesis, CCD-986sk human dermal fibroblasts cells were treated with PQQ at 100, 250, and 500 μM. Procollagen Type I C-peptide levels were quantified using ELISA and converted to the amount of collagen relative to the total protein amount of each sample to determine the intracellular collagen production rate. L-ascorbic acid 284 μM was used as a positive control. At PQQ concentrations of 100, 250, and 500 μM, collagen production rates were 134.5 ± 9.4%, 138.4 ± 4.9%, and 68.7 ± 3.3%, respectively, while L-ascorbic acid 284 μM, the positive control, showed 126.1 ± 3.2%. PQQ at 100 and 250 μM significantly increased collagen production compared with the untreated control, but at 500 μM, it showed a decrease in collagen synthesis. This suggests that high-dose PQQ can inhibit collagen production or inducing cytotoxicity. In summary, the highest collagen production value was observed at 250 μM among the tested concentrations, suggesting its potential as a functional cosmetic ingredient for improving skin elasticity through collagen regeneration [19] (Fig. 4B). To determine whether the reduced collagen synthesis observed at high PQQ concentrations was related to cytotoxicity, a cell viability assay was performed in CCD-986sk human dermal fibroblasts (Fig. 4A). The treatment with 500 μM PQQ significantly reduced fibroblast viability, indicating cytotoxic effects at this concentration. These results suggest that the decrease in collagen synthesis observed at high PQQ concentrations is at least partly due to reduced cell viability.

### Cell viability and Melanin Inhibition

**Fig. 5:**
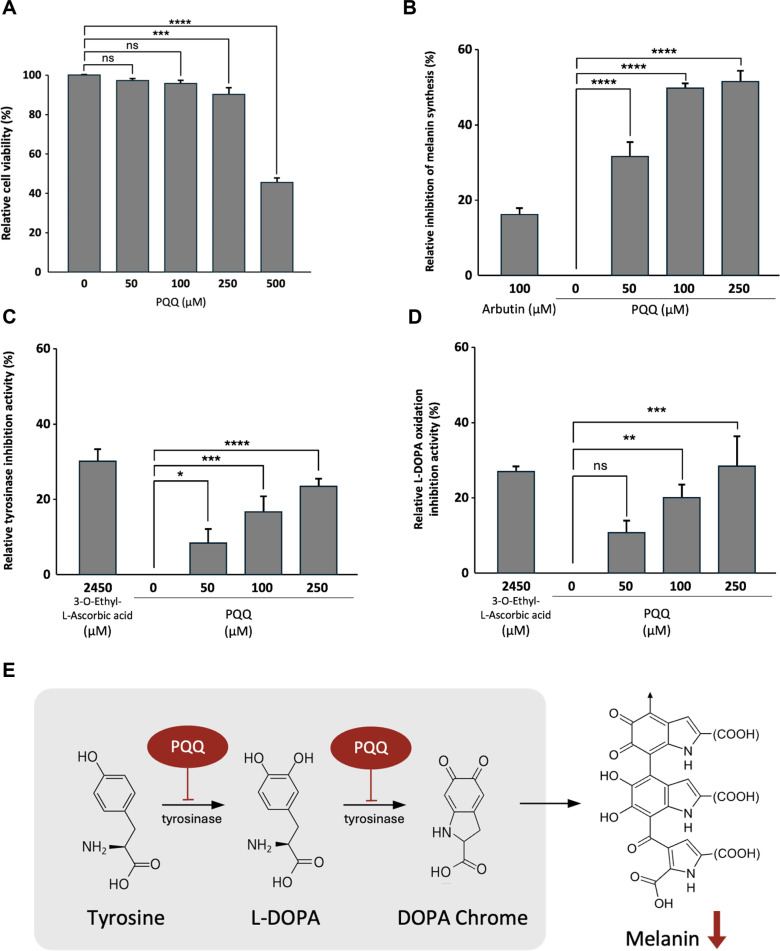
Effects of PQQ on cytotoxicity, tyrosinase activity, L-DOPA oxidation, and melanogenesis in B16-F10 cells. B16-F10 cells were treated with PQQ at various concentrations to evaluate its biological activities. (**A**) Cytotoxicity was assessed following treatment with PQQ at 50, 100, 250, 500, and 1,000 μM. (**B**) Tyrosinase inhibitory activity was determined using PQQ at 50, 100, and 250 μM (dissolved in distilled water). (**C**) L-DOPA oxidation inhibition was evaluated in samples treated with PQQ at 50, 100, and 250 μM. (**D**) Melanin production was measured in B16-F10 cells treated with PQQ at 50, 100, and 250 μM to assess its anti-melanogenic activity. Statistical significance was determined using one-way ANOVA followed by Dunnett’s test compared with the untreated control group (ns, not significant; *p < 0.05; **p < 0.01; ***p < 0.001; ****p < 0.0001). (**E**) Schematic diagram illustrating the inhibitory mechanism of PQQ on melanogenesis. The diagram summarizes the stepwise effects of PQQ on melanin synthesis: inhibition of tyrosinase activity (**B**), suppression of L-DOPA oxidation (**C**), and reduction of melanin production in B16-F10 cells (**D**), ultimately leading to decreased melanogenesis.

To evaluate the cytotoxicity and melanin inhibitory effect of PQQ, B16-F10 mouse melanoma cells were treated with the compound at concentrations ranging from 50 to 500 μM. Cell viability was assessed using the WST assay after 24 h of exposure, and intracellular melanin content was measured following 72 h treatment in the presence of α-MSH (100 nM), a known melanin inducer. In the cytotoxicity assay, cell viability remained above 90% at concentrations up to 250 μM, with values of 96.5 ± 1.5%, 95.6 ± 1.3%, and 89.9 ± 3.2% for 50, 100, and 250 μM, respectively. However, at 500 μM, cell viability decreased to 45.7 ± 1.9%, indicating dose-dependent cytotoxic effects at elevated concentrations. Therefore, concentrations up to 250 μM were selected for subsequent melanin inhibition assays (Fig. 5A).

In the intracellular melanin production inhibition test, PQQ showed melanin production inhibition rates of 31.8 ± 3.6%, 49.8 ± 1.4%, and 51.7 ± 2.9% at concentrations of 50, 100, and 250 μM, respectively, without cytotoxicity. Arbutin 100 μM, used as a positive control, showed inhibition activity of 16.5 ± 1.5%. As a result, PQQ inhibited melanin in a concentration-dependent manner within the concentration of 250 μM (Fig. 5B). This shows its potential as a whitening agent through melanin inhibition.

### Tyrosinase Inhibition Activity of PQQ

To investigate the potential of PQQ as a skin whitening agent, tyrosinase inhibition activity was investigated. PQQ solutions were prepared in distilled water and tested at final concentrations of 50, 100, and 250 μM. The results of the analysis showed that tyrosinase inhibition was 8.2 ± 3.9%, 17.1 ± 3.7%, and 23.0 ± 2.5% at PQQ concentrations of 50, 100, and 250 μM, respectively. The positive control, 3-O-ethyl ascorbic acid (2450 μM), showed an inhibition effect of 30.1 ± 3.1% (Fig. 5C). PQQ showed a concentration-dependent inhibition pattern, indicating that PQQ inhibits melanin biosynthesis by targeting tyrosinase activity, suggesting its potential as a functional cosmetic whitening ingredient.

### Inhibitory Effect of PQQ on L-DOPA Oxidation

To investigate the potential of PQQ as a skin whitening agent, its ability to inhibit L-DOPA oxidation was examined. PQQ was dissolved in distilled water and treated at final concentrations of 50, 100, and 250 μM, and 2450 μM of 3-O-ethyl ascorbic acid was used as a positive control. The oxidation inhibition activity was 10.5 ± 3.4%, 19.9 ± 3.5%, and 28.4 ± 7.8% at the concentrations of 50, 100, and 250 μM of PQQ, respectively, and the positive control, 3-O-ethyl ascorbic acid, showed 29.9 ± 1.2% (Fig. 5D). These results suggest that PQQ inhibited L-DOPA oxidation in a concentration-dependent manner, and that this inhibitory activity may help whiten skin by regulating melanin biosynthesis.

## Discussion

This study aimed to investigate the effects of mutant strain and optimized fermentation conditions on bacterial growth and PQQ production. Furthermore, the biological functions of microbially fermented PQQ were evaluated in terms of skin health and its potential use in the field of functional cosmetics.

The comparative growth analysis results between *H. denitrificans* wild and mutant strains clearly showed that the UV-induced mutant strain (NPP-230) achieved significantly higher biomass than the wild strain under both normal and optimized conditions. The absorbance (OD_600_) value of the mutant strain cultured in the optimized medium reached 4.252 ± 0.334, which was 8.8-fold higher than that of the wild strain under the same conditions and 39.4-fold higher than that of the wild strain in the normal medium. In this study, this significant increase may be attributed to the improved metabolic ability acquired through the mutation and the favorable medium composition including increased trace elements and carbon/nitrogen sources. These results suggest that genetic modification and environmental optimization can synergistically enhance microbial growth and metabolite biosynthesis [20].

PQQ production also showed a similar remarkable trend. The wild strain produced 3.06 ± 0.12 μM of PQQ in the normal medium, while the mutant strain reached 28.56 ± 10.19 μM under the optimized conditions, which was approximately 9.3-fold higher. Previous studies suggest that the enhanced PQQ production observed in the mutant strain may be attributed to a combination of UV-NTG mutagenesis and adaptive laboratory evolution under high methanol and oxidative stress conditions. Previous studies have shown that such treatments can induce beneficial mutations in genes related to methanol metabolism and oxidative stress response, such as formate-tetrahydrofolate ligase (ftfL) and thiamine biosynthesis protein C (thiC), ultimately promoting increased PQQ biosynthesis [17, 21-23].

On the other hand, the optimized medium slightly decreased the PQQ production of the wild strain, which may be due to the fact that the native metabolic pathway of the wild strain may not efficiently utilize the enhanced medium composition, unlike the mutant strain with enhanced expression or activity of PQQ biosynthetic genes (*e.g.*, pqqABCDE). These observations suggest the importance of adjusting the culture conditions according to the strain genotype to maximize yield.

The effects of biologically produced PQQ on skin physiology were also evaluated. PQQ treatment at concentrations of 100 and 250 μM significantly increased collagen synthesis in CCD-986sk fibroblasts, with 250 μM treatment showing the highest stimulation rate of 138.4 ± 4.9%, which surpassed L-ascorbic acid (126.1 ± 3.2%). However, collagen synthesis was decreased at 500 μM treatment, suggesting the possibility of a biphasic effect or cytotoxic stress at high doses. The decrease in collagen synthesis observed at the highest PQQ concentration (500 μM) initially suggested a potential inhibitory effect. However, cytotoxicity analysis demonstrated that high-dose PQQ significantly reduced fibroblast viability, indicating that the reduced collagen production was primarily associated with cytotoxic effects rather than direct inhibition of collagen biosynthesis. These findings highlight the importance of concentration-dependent effects when evaluating the biological activity of PQQ. These results suggest that PQQ can enhance skin elasticity through upregulation of collagen biosynthesis [19].

Cytotoxicity and melanin inhibition assays using B16-F10 murine melanoma cell line showed that cell viability was maintained at more than 89.9% at concentrations up to 250 μM, and also showed a dose-dependent melanin inhibition effect. At 250 μM concentration, melanin synthesis was inhibited by 51.7 ± 2.9%, which was significantly higher than that of the positive control (arbutin 16.5 ± 1.5%). In addition, PQQ showed a concentration-dependent inhibition effect on tyrosinase and L-DOPA oxidation, with a maximum inhibition rate of 23.0 ± 2.5% and 28.4 ± 7.8%, respectively. These results suggest that PQQ can downregulate melanin production by inhibiting the enzymatic activity of tyrosinase and by reducing the oxidative substrates required for melanin polymerization (Fig. 5E). This dual action supports the potential use of PQQ as a skin whitening agent in cosmetic formulations [24-26]. While this study demonstrated that PQQ significantly inhibits melanin synthesis by reducing melanin content, tyrosinase activity, and L-DOPA oxidation, it is important to note that the evaluation was limited to the biosynthetic phase of melanogenesis. Melanin deposition in the skin is ultimately determined not only by synthesis within melanocytes but also by the transport of melanosomes to surrounding keratinocytes [27]. Since melanosome transfer is a critical step in determining visible pigmentation [28], the absence of an assessment of melanin transport represents a limitation of our current work. Future studies are required to investigate whether PQQ also affects melanosome transfer using established assays such as the melanosome transfer assay. This would provide a more comprehensive understanding of PQQ’s potential as a skin-whitening agent. From a biochemical perspective, the redox-active orthoquinone structure of PQQ is likely to contribute to both antioxidant and anti-melanogenic functions. Unlike membrane-bound CoQ10, PQQ is water-soluble and can easily penetrate into the intracellular space, providing a broad range of protective effects against reactive oxygen species (ROS). Its unique ability to undergo repeated redox cycles without degradation may contribute to its sustained activity in biological systems [29].

This study demonstrates enhanced PQQ production and its bioactivity *in vitro*; however, several limitations should be acknowledged. First, the biological effects of PQQ were evaluated exclusively using *in vitro* models, and *in vivo* validation was not performed, which limits the direct extrapolation of these findings to complex biological systems. Future studies employing animal or 3D human skin models will be necessary to further confirm the efficacy and safety of PQQ. Second, although whole-genome sequencing of the mutant strain identified potential mutations related to methanol metabolism and oxidative stress response (e.g., ftfL and thiC), the precise molecular mechanisms responsible for the enhanced PQQ production were not functionally validated. Ongoing and future transcriptomic and integrative genomic analyses are expected to elucidate the regulatory and metabolic networks underlying this phenotype. In addition, while intracellular ROS measurements could have provided deeper mechanistic understanding into the antioxidant role of PQQ in collagen regulation, such assays were not conducted in the present study. The primary focus of this work was to assess the functional outcomes of PQQ treatment, particularly collagen production, rather than to comprehensively dissect the upstream signaling pathways involved. Future investigations incorporating ROS analysis and pathway-level studies will further clarify the mechanistic basis of the biological effects of PQQ.

Taken together, the data from this study provide evidence that UV-mutated *H. denitrificans* NPP-230 can be utilized as an efficient biological source of PQQ when cultured under the optimized conditions. Furthermore, the purified compound exhibits beneficial effects on skin physiology, particularly in promoting collagen synthesis and inhibiting melanin production, which are key targets in anti-aging and whitening cosmetics. These results demonstrate the potential of microbial fermentation as a sustainable PQQ production and suggest new opportunities for its commercial application as a multifunctional cosmetic ingredient.

## Supplemental Materials

Supplementary data for this paper are available on-line only at http://jmb.or.kr.



## Figures and Tables

**Table 1:** Media composition normal medium (DSMZ 166) and optimized medium.