# Supplementary material for: Optimization of Culture Conditions for Pyrroloquinoline Quinone-Overproducing Mutant Hyphomicrobium denitrificans and Its Skin Bioactive Properties
Source: J Microbiol Biotechnol. 2026 May 8;36:e2603038. doi: 10.4014/jmb.2603.03038 (PMC13158751; doi:10.4014/jmb.2603.03038)

J. Microbiol. Biotechnol. 2026. 36: e2603038

https://doi.org/10.4014/jmb.2603.03038

Supplementary Tables and Figure

Table S1. Foods containing PQQ (more than 10 ng/g) [2, 3].

Group	Food	PQQ (ng/g or *ng/mL)
Vegetables	Parsley	34
	Potatoes	17
	Sweet potatoes	13
	Spinach	22
	Carrots	17
	Cabbage	16
	Green pepper	28
Legumes	Broad beans	18
	Fava beans	18
Fruits	Banana	13
	Kiwi	27
	Papaya	27
Other foods	Tofu (bean curd)	24
	Miso (bean paste)	17
	Fermented bean products (Natto)	61
Beverages	Coke	20*
	Green tea	29*
	Oolong tea	34*
	Bovine milk	17*

Table S2. HPLC conditions to quantify PQQ in *H. denitrificans* cultivation medium.

<table>
<tr><th>Parameter</th><th colspan="3">Condition</th></tr>
<tr><td>Column</td><td colspan="3">YMC-Triart C18
(particle size 5 μm, 4.6 x 250 mm)</td></tr>
<tr><td>Dilution solvent</td><td colspan="3">Distilled water</td></tr>
<tr><td>Detection (nm)</td><td colspan="3">250</td></tr>
<tr><td>Column Tem. (℃)</td><td colspan="3">30</td></tr>
<tr><td>Injection Volume (μL)</td><td colspan="3">10</td></tr>
<tr><td rowspan="7">Mobile phase</td><td>Time(min)</td><td>A*</td><td>B**</td></tr>
<tr><td>6</td><td>97</td><td>3</td></tr>
<tr><td>6.01</td><td>20</td><td>80</td></tr>
<tr><td>27</td><td>20</td><td>80</td></tr>
<tr><td>27.01</td><td>97</td><td>3</td></tr>
<tr><td>33</td><td>97</td><td>3</td></tr>
<tr><td colspan="3">* A: 15 mM phosphate buffer (pH 7.4)
** B: Acetonitrile</td></tr>
</table>

Table S3. PQQ treatment conditions to confirm its effect on collagen synthesis in human dermal fibroblast (CCD-986sk).

Test group	Substance name	Treatment concentration
Control	-	-
Positive control	L-ascorbic acid	50 μg/mL
Treatment	PQQ	100 μM
		250 μM
		500 μM

Table S4. PQQ treatment conditions for evaluating cell viability in B16-F10 mouse melanoma cells.

Group	Treatment concentration of PQQ (µM)
Control	-
Treatment	50
	100
	250
	500
	1,000

Table S5. PQQ treatment conditions for assessing melanin inhibition in B16-F10 mouse melanoma cells.

**Group**	α-MSH (100 nM)	**Substance name**	**Substance treatment concentration (μM)**
Negative control	+	-	-
Positive control	+	Arbutin	100
Treatment	+	PQQ	50
			100
			250

Table S6. (A) Experimental groups for tyrosinase inhibition assay and **(B)** sample preparation.

A

Group		Substance name	Treatment concentration
a	Negative control	PBS	-
a'			
b	Positive control	3-O-Ethyl-L-ascorbic acid	500 μg/mL
b'			
b	Treatment	PQQ	50, 100, and 250 μM
b'			

B

Test group	50 μL	50 μL	10 μL
a	Tyrosinase 0.1 U/mL	Tyrosine 1 mM	PBS
b			Sample

Test group	50 μL	50 μL	10 μL
a'	Tyrosinase 0.1 U/mL	PBS	PBS
b'			Sample

a) tyrosinase 50 μL + L-tyrosine 50 μL + PBS 10 μL
a′) tyrosinase 50 μL + PBS 50 μL + PBS 10 μL
b) tyrosinase 50 μL + L-tyrosine 50 μL + PQQ or positive control 10 μL
b′) tyrosinase 50 μL + PBS 50 μL + PQQ or positive control 10 μL

Table S7. (A) Experimental groups for L-DOPA oxidation inhibition assay and **(B)** sample preparation.

A

Group		Substance name	Treatment concentration
a / a'	Negative control	PBS	-
b / b'	Positive control	3-O-Ethyl-L-ascorbic acid	500 µg/mL
b / b'	Treatment	PQQ	50, 100, and 250 µM

B

Test group	50 µL	50 µL	10 µL
a	Tyrosinase	L-DOPA	PBS
b	0.1 U/mL	0.6 mM	Sample

Test group	50 µL	50 µL	10 µL
a'	Tyrosinase	PBS	PBS
b'	0.1 U/mL		Sample

a) tyrosinase 50 µL + L-DOPA 50 µL + PBS 10 µL
a′) tyrosinase 50 µL + PBS 50 µL + PBS 10 µL
b) tyrosinase 50 µL + L-DOPA 50 µL + PQQ or positive control 10 µL
b′) tyrosinase 50 µL + PBS 50 µL + PQQ or positive control 10 µL

Fig. S1. Linear regression model to evaluate the relationship between OD_{600} and CFU/mL using adjusted log_{10} (CFU/mL) values. The data points in the graph are the results obtained from three independent experiments (R^2=0.975).

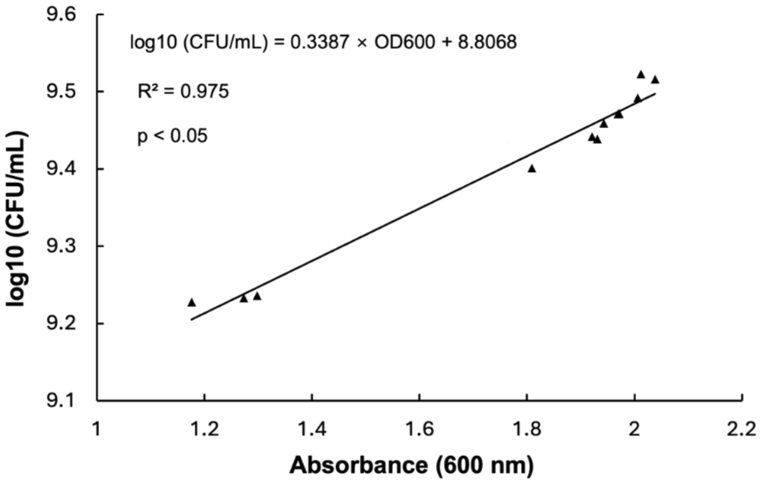

Supplement: Supplementary file 1 [file jmb-36-e2603038-supple.pdf]
